# Impact of Radiologist Experience on AI Annotation Quality in Chest Radiographs: A Comparative Analysis

**DOI:** 10.3390/diagnostics15060777

**Published:** 2025-03-19

**Authors:** Malte Michel Multusch, Lasse Hansen, Mattias Paul Heinrich, Lennart Berkel, Axel Saalbach, Heinrich Schulz, Franz Wegner, Joerg Barkhausen, Malte Maria Sieren

**Affiliations:** 1 Department of Radiology and Nuclear Medicine, UKSH, 23538 Lübeck, Germany; lennart.berkel@uksh.de (L.B.); franz.wegner@uksh.de (F.W.); malte.sieren@uksh.de (M.M.S.); 2 EchoScout GmbH, 23562 Lübeck, Germany; lasse@echoscout.ai; 3 Institute of Medical Informatics, University of Lübeck, 23538 Lübeck, Germany; mattias.heinrich@uni-luebeck.de; 4 Philips Innovative Technologies, 22335 Hamburg, Germany; axel.saalbach@philips.com (A.S.); heinrich.schulz@philips.com (H.S.)

**Keywords:** annotation quality, interreader comparison, chest radiograph, AI research

## Abstract

**Background/Objectives**: In the burgeoning field of medical imaging and Artificial Intelligence (AI), high-quality annotations for training AI-models are crucial. However, there are still only a few large datasets, as segmentation is time-consuming, experts have limited time. This study investigates how the experience of radiologists affects the quality of annotations. **Methods**: We randomly collected 53 anonymized chest radiographs. Fifteen readers with varying levels of expertise annotated the anatomical structures of different complexity, pneumonic opacities and central venous catheters (CVC) as examples of pathologies and foreign material. The readers were divided into three groups of five. The groups consisted of medical students (MS), junior professionals (JP) with less than five years of working experience and senior professionals (SP) with more than five years of experience. Each annotation was compared to a gold standard consisting of a consensus annotation of three senior board-certified radiologists. We calculated the Dice coefficient (DSC) and Hausdorff distance (HD) to evaluate annotation quality. Inter- and intrareader variability and time dependencies were investigated using Intraclass Correlation Coefficient (ICC) and Ordinary Least Squares (OLS). **Results**: Senior professionals generally showed better performance, while medical students had higher variability in their annotations. Significant differences were noted, especially for complex structures (DSC Pneumonic Opacities as mean [standard deviation]: MS: 0.516 [0.246]; SP: 0.631 [0.211]). However, it should be noted that overall deviation and intraclass variance was higher for these structures even for seniors, highlighting the inherent limitations of conventional radiography. Experience showed a positive relationship with annotation quality for VCS and lung but was not a significant factor for other structures. **Conclusions**: Experience level significantly impacts annotation quality. Senior radiologists provided higher-quality annotations for complex structures, while less experienced readers could still annotate simpler structures with satisfying accuracy. We suggest a mixed-expertise approach, enabling the highly experienced to utilize their knowledge most effectively. With the increase in numbers of examinations, radiology will rely on AI support tools in the future. Therefore, economizing the process of data acquisition and AI-training; for example, by integrating less experienced radiologists, will help to meet the coming challenges.

## 1. Introduction

In the rapidly evolving landscape of medical imaging and artificial intelligence, the development of robust AI models for chest radiograph interpretation is at the brink of entering clinical routine [1,2]. However, a bottleneck in this advancement is the quality of the annotations used to train these AI systems. Annotations serve as the foundational data from which AI models learn to recognize anatomy, foreign material, and diagnose pathologies [3]. On the one hand, the accuracy and reliability of these annotations are paramount, as they directly influence the effectiveness and trustworthiness of AI diagnostic tools [4,5]. On the other hand, annotations are time-consuming, and the availability of medical experts is often limited [6]. Although recent studies have shown that In-Image-Annotation enhances the performance of AI models [7,8], many approaches still rely on image labeling. Even in image labeling, a certain degree of interreader variability is found [9], especially in chest radiographs [10]. While some studies have already been conducted in which different levels of reader experience were mentioned in the methods section, further analysis regarding these different experience levels is often lacking [11,12].

In the few cases where such an analysis has been conducted, dedicated training for a certain task or topic not only led to better overall performance by the reader but also resulted in a smaller interreader variance [13,14].

Nevertheless, recent trends underscore the importance of diversified expertise in the annotation process [15]. While experienced radiologists bring a nuanced understanding to the task, readers with less experience, such as junior radiologists or trainees, may have better availability [16]. This dichotomy raises a pertinent question: How does the level of experience impact the quality of chest radiograph annotations, and in turn, the performance of AI models trained on these annotations [7]?

This paper aims to investigate the impact of radiologic experience on the quality of in-image-annotation in chest radiographs dedicated for AI training by analyzing annotations of several typical imaging findings by a spectrum of readers. 

## 2. Materials and Methods

### 2.1. Data Collection

Our dataset comprised 53 chest radiographs from distinct patients (gender distribution: 26 males, 27 females; average age: 68 ± 12 years). These images were randomly selected from the local Picture Archiving and Communication System archive and anonymized at the source to maintain patient confidentiality. During the study, all images handled were fully anonymized and neither of the researchers had access to any personal data of the persons the images belonged to.

The dataset exclusively includes in-bed anterior–posterior projection images acquired in the intensive care unit during February 2022. Typical acquisition parameters for these images were 90–109 kV and 0.8 mAs, source detector distance 1 m. Inclusion criteria were the presence of a pneumonic opacity, as confirmed by clinical examination and laboratory data, alongside three trained radiologists’ consensus. Additionally, 40 patients included in the study had a CVC in place.

### 2.2. Segmentation Process

For the segmentation task, we formed three teams, each comprising five readers but differing in their levels of medical expertise. Team MS (medical students with at least four years in university) included medical students who had undergone dedicated training with fifty chest radiographs, equipping them with basic knowledge and skills. The training included a short recap on basic anatomical and pathological findings in Chest Radiographs as well as technical training to ensure the correct application of the annotation tool. Team JP (Junior Professionals) consisted of residents from the radiology department, each with one to four years of practical experience. Team SP (Senior Professionals) was composed of senior radiologists, each with over five years of professional experience. A consensus annotation of three senior Radiologists, each with more than ten years of experience, served as a Gold Standard. Every team received a detailed introduction to the segmentation tool CVAT and a guide with instructions for segmenting the structures with image examples. Each reader received a 15 min training session with one of the authors of the study in which 20 example images were annotated. These images were not part of the later study data.

**Figure 1:**
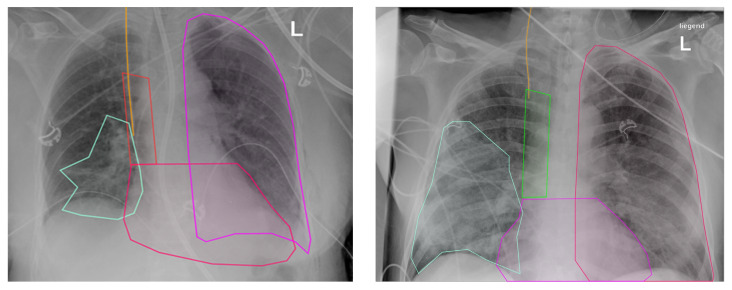
Annotated chest radiographs.

The annotation process was facilitated by the browser-based Computer Vision Annotation Tool (CVAT; 1.1b0, Intel, Santa Clara, CA, USA), which was integrated into our clinical network. This tool was adapted to enable DICOM-Annotation. Annotations were performed using polygon boxes or polygon lines, as illustrated in Figure 1. All images had to be annotated in one session without breaks and in the same order for every reader; the session was repeated after four weeks to assess for intrareader differences.

The annotated structures included:

1. Anatomical Structures: These were categorized based on their level of difficulty in terms of identification and annotation. The structures included were the left lung (easy), the heart (medium), and the superior vena cava (VCS) (hard).
2. Foreign Material: The CVC, if present, was annotated. In many cases, the CVC had to be distinguished from other tube-like foreign material, e.g., dialysis catheters. Twelve of the images showed a misplaced CVC.
3. Pulmonary Pathology: Pneumonic opacity was annotated. All participants were aware that all patients in the cohort were treated for pneumonia.

Annotated chest radiograph in the specialized CVAT annotation program. Each component, including the CVC, heart, lungs, VCS, and a pneumonic opacity in the right lung, is marked using polygonal boxes and lines. Left: female patient, 63 years. Right: male patient, 71 years.

### 2.3. Statistics

Statistics were performed using GraphPad Prism (GraphPad Prism 10.1.2, GraphPad Software, San Diego, CA, USA). The Hausdorff distance (HD) was calculated for the Central Venous Catheter (CVC), while the DICE coefficient (DSC) was computed for all other structures.

Statistical evaluation employed descriptive statistics (mean, median, standard deviation, range, and misses), calculated for each group across different structures. Misses were defined as a DSC of <0.25 or HD of >30 mm. The Shapiro–Wilk test was deployed to check for normal distribution, which was not given. To assess the significance of differences between groups, pairwise comparisons were therefore analyzed using the Mann Whitney U test. Inter- and intrareader consistency within each group was evaluated using the Intraclass Correlation Coefficient (ICC), providing insights into the reliability of annotations within each experience level. *p*-values less than 0.05 were considered statistically significant.

Moreover, we conducted a Spearman correlation and an Ordinary Least Squares (OLS) regression analysis to explore the relationship between the annotators’ experience [years] (independent variable) and the quality of their annotations for each structure separately [DSC or HD depending on the structure] (dependent variable).

To assess possible variances in annotation quality over the time of annotation (the whole dataset had to be annotated in one session without breaks), a time-series analysis was performed. Performance metrics for lung, heart, and VCS were averaged across all reader groups for each image, creating visual plots to observe trends. Then, we applied the Mann–Kendall trend test to statistically ascertain these trends’ significance.

## 3. Results

Detailed descriptive statistics can be found in Table 1.

**Figure 2:**
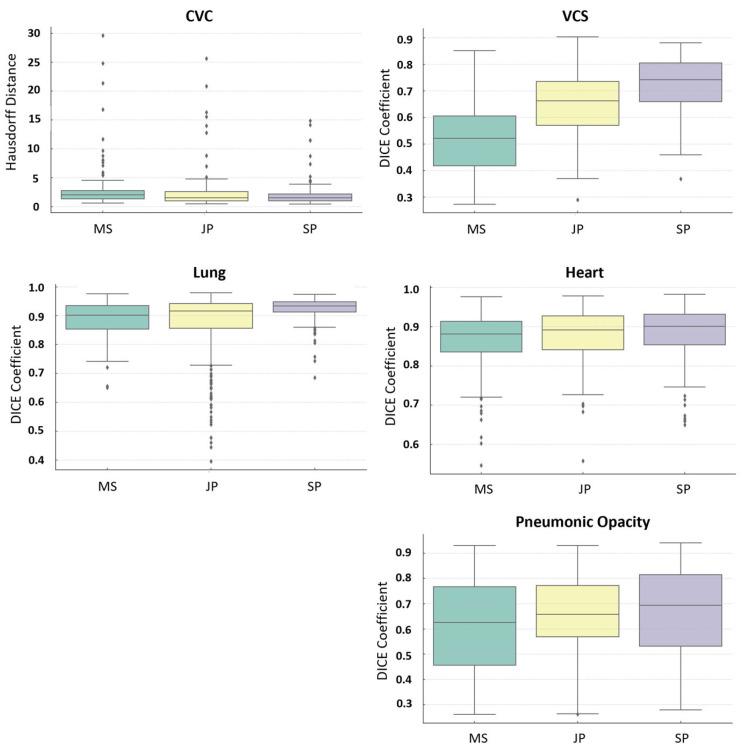
Distribution of segmentation quality.

Our analysis revealed notable differences in annotation quality among the groups. The SP group generally exhibited superior or comparable performance across most structures, with fewer misses. In contrast, the least experienced group, MS, showed greater variability and a higher number of misses, particularly when annotating pneumonic opacities. The intermediate group, JP, often fell between the other two groups in terms of performance. Figure 2 provides an overview of the agreement in annotations between the different groups, showing great agreement in, for example, the lung and the CVC while structures like the VCS showed greater variance.

When assessing specific structures, significant differences emerged, as follows:

1. CVC: Significant differences were noted between the group MS and JP, as well as the MS and SP groups.
2. VCS: All pairs of groups exhibited significant differences in their annotations.
3. Heart Structure: A significant difference was noted only between the MS and SP groups.
4. Lung Structure: Significant differences were found between the JP and SP groups, as well as the MS and SP groups.
5. Pneumonic Opacity Structure: Significant differences were observed between the MS and JP groups, and the MS and SP groups.

The figure presents a series of boxplots comparing the distribution of the Dice Coefficient and Hausdorff Distance for annotations across three groups labeled MS, JP and SP. The Dice Coefficient boxplots illustrate the agreement between annotators on the VCS, heart, pneumonic opacity, and lung annotations, with higher values indicating better agreement. The Hausdorff Distance boxplot indicates the agreement within the CVC annotations, where lower values represent closer agreement between annotators. Each boxplot details the median (central line), interquartile range (box edges), and outliers (individual points).

**Figure 3:**
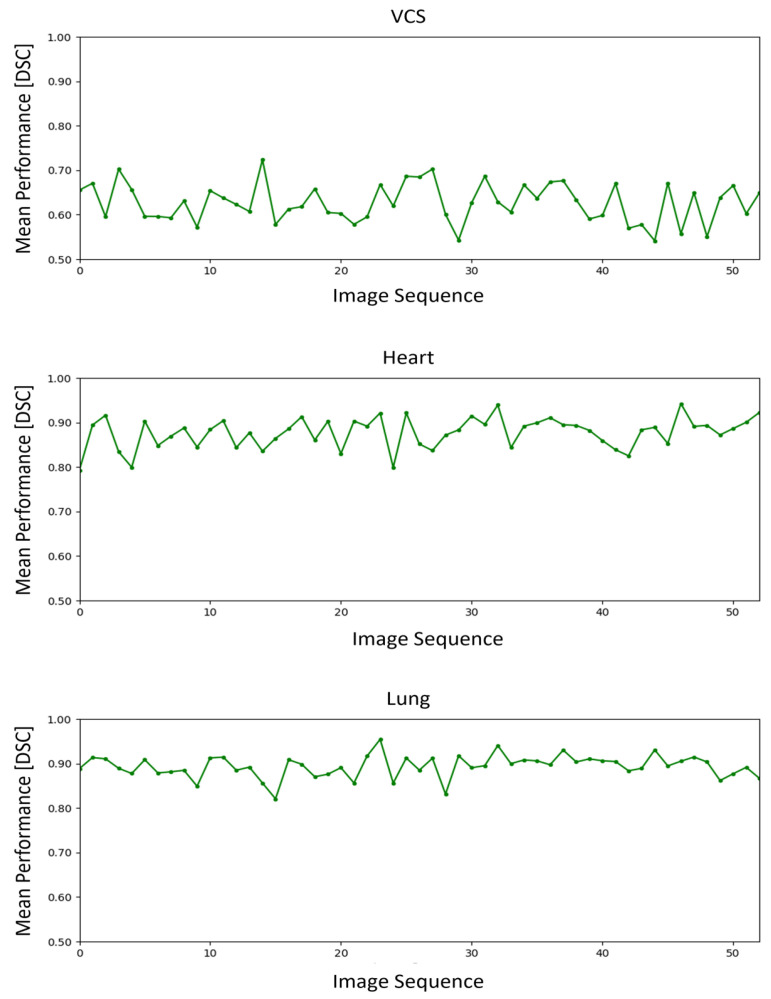
Overall segmentation quality of different images.

As seen in Figure 3, some variance could be seen through the sequence of 53 radiographs, demonstrating that not only the knowledge of the annotator but also the quality of the image might contribute to the quality of annotation. Complicating factors identified included deficiencies in image quality, such as malrotation or incorrect exposure, as well as pathologies masking anatomical boundaries, e.g., pleural effusion or atelectasis. However, our analysis showed that more experienced participants clearly better obtained results on images that seemed to have a higher difficulty than the less experienced, indicating that a nuanced understanding of the image and its flaws can at least partially compensate for image quality deficiencies.

Inter- and intrareader consistency, measured by the Intraclass Correlation Coefficient (ICC), varied across structures and was the worst for the CVC and relatively low for Pneumonic Opacities. In both, ICC improved markedly with experience. Across all other structures, the ICC was generally good to excellent within each group (Table 2).

The figure contains three line-graphs displaying the annotation quality, as measured by the Dice similarity coefficient averaged across all 15 readers for different structures in a sequence of 53 chest X-ray images. The top graph for the VCS, the middle for the heart, and the bottom for the lungs, all show fluctuations in annotation quality across the images but no notable decline over the image sequence.

### 3.1. Correlation and Regression Analysis

The Spearman correlation analysis indicated that CVC had a weak negative correlation with greater experience, VCS had a moderate positive correlation, and the heart, lung, and pneumonic opacity showed weak positive correlations. All correlations were statistically significant. The regression analysis conducted on different structures in relation to readers’ years of experience yielded varying results. Only years of professional expertise were counted; therefore, medical students were considered “0 years”. For CVC, heart, and pneumonic opacity, the years of experience had no statistically significant impact on annotation quality. Conversely, for the VCS and lung structures, the analysis revealed a statistically significant, albeit small, positive relationship between years of experience and annotation quality (Table 3).

### 3.2. Time-Series Analysis

In the time-series analysis, the lung and heart structures showed a non-statistically significant (a = 0,05) increasing trend (Tau: 0.07, *p*-value: 0.48 for lung; Tau: 0.15, *p*-value: 0.11 for heart), and the VCS structure exhibited a non-statistically significant decreasing trend (Tau: −0.05, *p*-value: 0.57).

## 4. Discussion and Conclusions

Our study examines the effect of radiologist experience levels on the quality of chest radiograph annotations for AI training. We discovered that more experienced radiologists generally provided higher-quality annotations, particularly for complex structures. Interestingly, our findings also suggest that less experienced personnel can effectively participate in annotating simpler structures, achieving satisfactory results.

Segmentations are acknowledged as enhancing AI performance in radiographic analysis [7]; however, focused studies on annotation quality in radiographs are scarce. A study by Thelle A et al. demonstrates robust intra- and interreader reliability in the context of pneumothorax measurements in radiographs, with intrarater reliability at 0.98 (95% CI lower limit 0.96) and interrater reliability at 0.95 (95% CI lower limit 0.93) [17]. Despite these high reliability scores, they may not encompass the breadth of the annotation diversity required for holistic AI training. Rajaraman et al.’s research delves into interreader and intrareader variability in chest radiograph interpretations for COVID-19, noting accuracy, sensitivity, and specificity in the range of 0.58–0.78 [18]. Although the study does not directly evaluate annotation quality, it underscores that observer variability in radiographs tends to be greater than variability from the same observer over time, which is confirmed by our study. However, a study that systematically evaluates annotations in radiographs is lacking.

**Figure 4:**
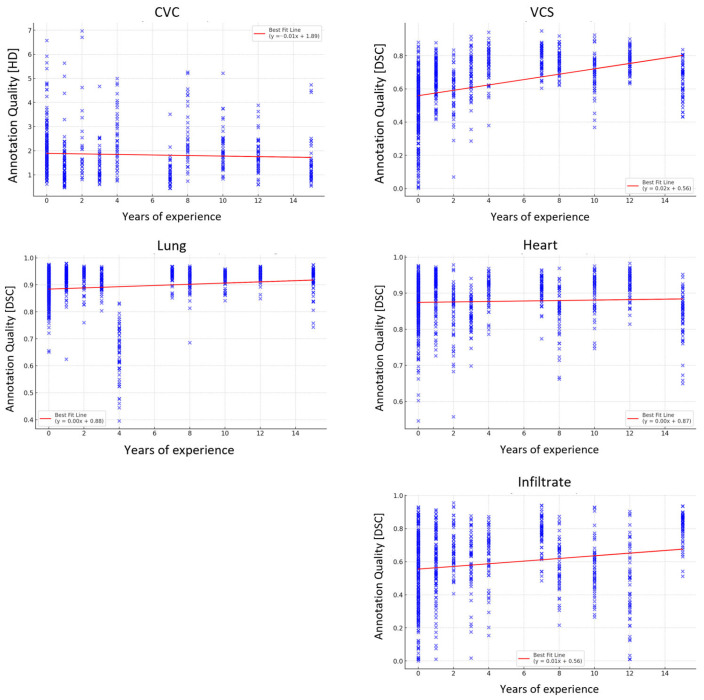
Correlation of experience with segmentation quality.

As expected, the overall quality of annotations increased with the readers’ experience; however, there were findings that deviated from this outcome. Somewhat intuitive, the differences were most pronounced when annotating structures that cannot be delineated well in radiographs, such as the VCS and pneumonic opacities. The most frequent errors occurred when annotating pneumonic opacities, which is a challenging task due to the inherent limitations of radiography. Again, these were less pronounced for the experienced readers. For more straightforward structures like the heart and the lung, the differences were far less distinct. However, more experienced readers did not always outperform the less experienced readers. For example, a single reader in the junior professional group did not adhere to the previously given instructions when annotating the lung and performed far worse than their peers, maybe reflecting some kind of overconfidence. Furthermore, the by-far most experienced reader presented only an average performance compared to the other junior and senior professionals for most structures, as illustrated in Figure 4. The regression analysis for CVC annotation even revealed a drop in performance with increased experience over all readers. This suggests that other factors might be at play. With tasks perceived as simple, there is a possibility that experienced readers may not engage with them as diligently. Additionally, the generally moderate annotation outcomes for the CVC suggest that such tasks might be approached with less precision, irrespective of the reader’s experience. The absence of a stimulating challenge could contribute to a decline in attentiveness. While our data did not demonstrate this effect, it could become apparent in analyses of larger datasets.

The provided figure displays scatter plots correlating years of experience with annotation quality for various medical imaging assessments: CVC, VCS, lung, heart, and pneumonic opacity, given as DSC or HD. The plots show data points with a line of best fit indicating the trend. There is a positive correlation between CVC and pneumonic opacity, with accuracy and quality increasing with experience, while the VCS, lung, and heart plots show less distinct trends.

The significant regression results for VCS and pneumonic opacities imply that experience plays a role, but its overall impact, especially for simple structures, is limited, and other factors might be crucial. This resonates with the findings of Wang et al., who noted that while experience is important, adaptability to annotation tools and specific training for AI applications can also significantly impact annotation quality [19]. It is understood that with structured training and guidance, the quality of the annotations can be enhanced, even among less experienced annotators. This aspect is underlined by the high consistency across all experience levels, as evidenced by the ICC, indicating a certain robustness in annotations [20]. Another quality guarantor that can further enhance robustness would be a structured review process, which was not investigated in this study.

The limitations of this study primarily stem from its scope and methodology. A key limitation is the sample size and diversity; with a relatively small and homogenous group of radiologists, the results may not be generalizable to a broader population. The study’s reliance on specific types of chest radiographs also restricts the applicability of its findings to other types of medical imaging. Additionally, the subjective nature of radiographic annotation introduces an inherent bias, which may have influenced the results. The study also did not account for variables like time pressure or fatigue, which can affect annotation accuracy.

Future work could focus on other possible influences on annotation quality, such as time pressure or fatigue. Whether annotation quality decreases significantly during a longer session of continuous annotation remains to be explored. While our study investigates the influence of radiologic experience on annotation quality in a short session, the influence of medical expertise on potential resilience against declining quality over time was not addressed.

While this study has shown that readers with little radiologic expertise can annotate simpler structures with satisfactory accuracy, future work could evaluate the actual impact of integrating amateurs into the process of data segmentation. For example, a study could be conducted comparing the performance of three AI models, one with a large dataset exclusively annotated exclusively by amateurs, one with a relatively small dataset of high-quality annotations performed by experts, and a third one created using a mixed expertise approach, with the number of data points falling in between those of the other datasets. This could serve as a benchmark for future AI research.

However, this study only focuses on chest radiographs. The inherent limitations of this modality might have influenced the results. The transferability of the results to other modalities of radiologic data, such as CT or MRI, needs to be addressed in future studies.

In conclusion, this study highlights the significant impact of experience on the accuracy and reliability of chest radiograph annotations, which are potentially critical for training AI models in medical diagnostics. The findings indicate that while experienced radiologists provide superior annotations, especially for complex structures, less experienced personnel can still contribute effectively to simpler annotations, thereby enabling the well-experienced to utilize their knowledge most effectively. The high intrareader consistency suggests that with appropriate instructions and training, even beginners can achieve a reliable annotation performance. Overall, the study advocates for a balanced approach that combines experience, targeted training, and technological support to enhance the quality and reliability of medical imaging annotations.

## Figures and Tables

**Table 1:** Descriptive statistics of main findings.

Structure	Groups	Mean	Std. Dev.	Min.	0.25	Median	0.75	Max.	Misses [%]
CVC HD [mm]	MS	22.43	56.96	0.62	1.48	2.26	7.82	524.18	18
	JP	26.39	95.17	0.47	1.04	1.63	3.34	554.16	12
	SP	17.41	76.72	0.4	1.13	1.67	2.51	556.18	4
VCS DICE	MS	0.48	0.20	0.0002	0.35	0.52	0.63	0.88	1.8
	JP	0.67	0.13	0.069	0.60	0.68	0.76	0.94	0.4
	SP	0.73	0.10	0.368	0.68	0.74	0.80	0.95	0
Heart DICE	MS	0.867	0.068	0.546	0.836	0.881	0.913	0.977	0
	JP	0.880	0.064	0.558	0.842	0.892	0.928	0.978	0
	SP	0.885	0.064	0.650	0.854	0.901	0.932	0.983	0
Lung DICE	MS	0.890	0.057	0.651	0.853	0.902	0.935	0.976	0
	JP	0.866	0.123	0.396	0.856	0.916	0.942	0.979	0
	SP	0.925	0.038	0.685	0.913	0.934	0.948	0.974	0
Pneumonic Opacity DICE	MS	0.516	0.246	0.00004	0.339	0.560	0.711	0.931	16.4
	JP	0.621	0.181	0.010	0.524	0.647	0.748	0.956	5.2
	SP	0.631	0.211	0.010	0.503	0.660	0.806	0.941	4.8

Comprehensive statistical summary of the Dice Coefficient and Hausdorff Distance for annotations on different structures: CVC, VCS, heart, lung, and pneumonic opacity, across three different groups labeled MS, JP, and SP.

**Table 2:** Intraclass correlation and intrareader consistency.

Structure	Groups	Interreader	Intrareader
CVC HD [mm]	MS	0.38	0.81
	JP	0.41	0.84
	SP	0.61	0.87
VCS DICE	MS	0.90	0.85
	JP	0.86	0.80
	SP	0.85	0.82
Heart DICE	MS	0.867	0.88
	JP	0.880	0.88
	SP	0.885	0.91
Lung DICE	MS	0.90	0.92
	JP	0.92	0.94
	SP	0.98	0.93
Pneumonic Opacity DICE	MS	0.45	0.61
	JP	0.52	0.72
	SP	0.66	0.77

The table shows the intraclass correlation coefficient (ICC) values for interreader and intrareader agreement among MS, JP, and SP for different thoracic structures. Higher ICC values indicate better reliability. Dice coefficients for the VCS, heart, and lungs show high reliability, especially among SPs. The CVC has moderate reliability, and pneumonic opacity shows the lowest reliability, particularly in interreader agreement.

**Table 3:** Correlation of segmentation quality with experience.

Structure	R-Squared	Correlation (Experience)	*p*-Value	Spearman Coefficient	*p*-Value
CVC	0.001	n.s.	0.34	−0.150	<0.001
VCS	0.136	0.0120	<0.05	0.545	<0.001
Heart	0.015	0.0018	<0.05	0.081	<0.05
Lung	0.001	n.s.	0.89	0.180	<0.001
Pneumonic Opacity	0.003	n.s.	0.12	0.173	<0.001

The table shows statistical analyses of the relationship between annotation quality for chest structures and annotator experience. The VCS and heart have a significant positive correlation with experience, while CVC shows a non significant, but low negative correlation. All Spearman coefficients are statistically significant, indicating a varying degree of correlation with experience across the different structures. However, the regression analysis confirms this finding only for the lung and the VCS, with a relatively small impact. The confidence interval was 95% (a = 0.05).

## Data Availability

To maintain patient confidentiality, access to the study data will only be granted by the authors upon justified request.

## Abbreviations

The following abbreviations are used in this manuscript:

HD	Hausdorff Distance
DSC	Dice Coefficient
ICC	Intraclass Correlation Coefficient
OLS	Ordinary Least Squares (OLS)
VCS	Vena Cava Superior
MS	Medical Students
JP	Junior Professionals
SP	Senior Professionals
